# Efficacy and Safety of Donor Lymphocyte Infusion After Allogeneic Hematopoietic Stem Cell Transplantation in Pediatric Patients

**DOI:** 10.1111/ejh.70112

**Published:** 2026-01-05

**Authors:** Dinah Walther, Jana Ernst, Carola Wollenhaupt, Susan Wittig, Manuela Härtel, Grit Brodt, Till Milde, Bernd Gruhn

**Affiliations:** ^1^ Department of Pediatrics Jena University Hospital Jena Germany; ^2^ Comprehensive Cancer Center Central Germany (CCCG) Jena Germany; ^3^ Hopp Children's Cancer Center Heidelberg (KiTZ) Heidelberg Germany; ^4^ Clinical Cooperation Unit Pediatric Oncology German Cancer Research Center Heidelberg (DKFZ) Heidelberg Germany

**Keywords:** donor chimerism, donor lymphocyte infusion, graft‐versus‐host disease, graft‐versus‐leukemia effect, hematopoietic stem cell transplantation

## Abstract

This retrospective study evaluates the efficacy and safety of donor lymphocyte infusion (DLI) after allogeneic hematopoietic stem cell transplantation (HSCT) in children. We describe the long‐term use of preemptive, prophylactic, and therapeutic DLI with a gradual dose increase in half‐log increments. Under close monitoring, we increased the DLI dose only in patients that did not show signs of graft‐versus‐host disease (GVHD). In the preemptive cohort, 10 of 12 patients (83%) with minimal residual disease (MRD) positivity remained relapse‐free. Among 11 patients with genetic diseases and mixed chimerism, nine (82%) responded to preemptive DLI. Six patients (100%) of the prophylactic cohort with a very high risk of relapse had a successful outcome without relapse or GVHD. Three of the five patients (60%) of the therapeutic cohort were successfully treated with DLI. We observed acute GVHD (grade I and II) in only two patients (6%). The results of our study indicate that the long‐term use of DLI is a promising strategy and can effectively prevent relapse, graft rejection, and even cure relapse. The observed low rate of GVHD may be attributed to the gradual dose increase. Therefore, we consider DLI a safe and effective therapeutic option.

## 1 Introduction

Allogeneic hematopoietic stem cell transplantation (HSCT) is a potentially curative therapy for pediatric patients with high‐risk malignancies and genetic diseases [1, 2]. In HSCT, hematopoietic stem cells from a healthy donor are transferred to the patient to reconstitute hematopoiesis and restore a functional immune system. However, relapse and graft rejection remain major reasons for treatment failure. In general, patients who experience relapse after HSCT have a dismal prognosis, with limited therapeutic options and poor long‐term survival rates [3, 4].

To improve outcomes, donor lymphocyte infusion (DLI) has emerged as a promising strategy to boost the graft‐versus‐leukemia (GVL) effect or graft‐versus‐tumor (GVT) effect and thereby reduce the risk of relapse in patients with malignant disease [5]. It is also an established immunotherapeutic option that is known to increase donor chimerism and prevent imminent graft failure after HSCT in patients with genetic disease. Data from various studies have demonstrated that DLI can effectively be used in patients who show early signs of relapse such as persisting minimal residual disease (MRD) or mixed chimerism (MC) [6].

Depending on the clinical setting, DLI can be administered as preemptive DLI to counter early signs of relapse or graft rejection, as prophylactic DLI in patients with a very high risk of relapse, or as therapeutic DLI in case of manifest hematological relapse. However, the efficacy of DLI treatment is limited by the induction of graft‐versus‐host disease (GVHD), the most common and adverse DLI effect. GVHD is a severe and potentially life‐threatening donor‐derived immune response against recipient tissues. It primarily affects the gastrointestinal tract, skin, and liver, and can manifest as an acute or chronic form potentially leading to severe long‐term complications [7].

Given these risks, it is essential to identify the safest possible approach for DLI administration to minimize adverse effects such as GVHD. Although there is a growing body of practical recommendations and standardized protocols for the use of DLI in adults, comparable guidelines for pediatric patients are still lacking. Critical aspects such as the optimal timing, dosing, and frequency of DLI administration to maximize therapeutic benefit while minimizing the risk of GVHD remain subjects of ongoing debate. To contribute to this discussion, we present our single‐center experience with the long‐term use of DLI in pediatric patients after allogeneic HSCT, evaluating efficacy and safety across prophylactic, preemptive, and therapeutic cohorts.

## 2 Patients and Methods

### 2.1 Study Population

The study was approved by the local institutional review board (2024‐3396). We included 34 pediatric patients (20 male, 14 female) who received DLI after HSCT between 01.07.2006 and 08.02.2024 at the Department of Pediatrics, Jena University Hospital, Jena, Germany in a single‐center retrospective cohort study. The median age at first DLI administration was 6.5 years (range: 2 months–18 years). Thirty‐one patients received allogeneic HSCT from a matched unrelated donor with a minimum of nine out of 10 HLA molecular match. Three patients received haploidentical HSCT. The underlying diseases were genetic diseases (*n* = 11), acute lymphoblastic leukemia (ALL) (*n* = 10), acute myeloid leukemia (AML) (*n* = 6), juvenile myelomonocytic leukemia (JMML) (*n* = 2), chronic myeloid leukemia (CML) (*n* = 1), myelodysplastic syndrome (*n* = 1), and solid tumors (*n* = 3). Based on the reasons for DLI administration we defined a preemptive cohort (*n* = 23), a prophylactic cohort (*n* = 6), and a therapeutic cohort (*n* = 5). Patients of the preemptive cohort were divided into those receiving DLI because of MRD reappearance in patients with hematological malignancies (*n* = 12) and those with MC in patients with genetic disease (*n* = 11). The prophylactic cohort included six patients with a very high risk of relapse. The therapeutic cohort consisted of four patients suffering from hematological relapse and one patient who was affected by recurrent autoimmune hemolytic crises. In case of multiple HSCT, we took only the first HSCT and following doses of DLI into consideration. Six Patients were excluded due to the fact that complete remission (CR) was not achieved at the time of HSCT and one patient due to missing data. No patients were excluded based on other criteria like gender, disease type or conditioning regimen. Patient characteristics are shown in the Table 1.

**TABLE 1:** Characteristics of patients (*n* = 34).

Characteristics	No. (%)
Sex	
Male	20 (58.8)
Female	14 (41.2)
Median age in years	6.5
Stem cell source	
Unrelated donor	31 (91.2)
Haploidentical donor	3 (8.8)
Human leukocyte antigen match	
Unrelated donor	
9/10 match	15 (44.1)
10/10 match	16 (47.1)
Haploidentical donor	
5/10	3 (8.8)
Reason for donor lymphocyte infusion	
Preemptive cohort	23 (67.6)
Detection of minimal residual disease	12
Mixed chimerism	11
Prophylactic cohort (high risk of relapse)	6 (17.7)
Therapeutic cohort	5 (14.7)
Hematological relapse	4
Recurrent autoimmune hemolytic crises	1
Diagnosis	
Genetic disease:	11 (32.4)
Mucopolysaccharidosis type II	3 (27.3)
Common variable immunodeficiency	2 (18.2)
Mucopolysaccharidosis type I	1 (9.1)
X‐linked lymphoproliferative disease	1 (9.1)
X‐linked adrenoleukodystrophy	1 (9.1)
Homozygous mutant for DCLRE1C gene	1 (9.1)
Reticular dysgenesis	1 (9.1)
Septic granulomatosis	1 (9.1)
Acute lymphoblastic leukemia	10 (29.4)
Acute myeloid leukemia	6 (17.7)
Juvenile myelomonocytic leukemia	2 (5.9)
Chronic myeloid leukemia	1 (2.9)
Myelodysplastic syndrome	1 (2.9)
Solid tumor	3 (8.8)

### 2.2 DLI

All patients received at least two doses of DLI from the same donor who provided the hematopoietic stem cells. We collected DLI samples either at time of HSCT or after the decision for DLI was made. Therefore, DLI was mostly administered as a cryopreserved sample. In several cases patients received freshly isolated cells with the first DLI dose. We started at a median DLI dose of 1.0 × 10^5^ CD3 cells/kg (range 1.0 × 10^4^–1.0 × 10^6^ CD3 cells/kg) in the preemptive cohort and 3.2 × 10^4^ × CD3 cells/kg (range 1.0 × 10^4^–1.0 × 10^5^ CD3 cells/kg) in the prophylactic cohort. In the therapeutic cohort, we started at a median DLI dose of 1.0 × 10^6^ CD3 cells/kg (range 1.0 × 10^5^–1.0 × 10^6^ CD3 cells/kg) except for the patient who was affected by recurrent autoimmune hemolytic crises where we started at 3.2 × 10^4^ CD3 cells/kg. In patients treated with DLI after haploidentical HSCT we administered a median DLI dose of 3.2 × 10^4^ CD3 cells/kg (range 1.0 × 10^4^–1.0 × 10^5^ CD3 cells/kg). We increased in half‐log increments to a maximum of 3.2 × 10^7^ CD3 cells/kg. DLI administration was started at a median time of 4 months (range 23–776 days) after HSCT, more precisely after a median time of 234 days (range 74–769 days) in patients with MRD in the preemptive cohort and 88 days (range 23–311 days) in patients with genetic disease in the preemptive cohort. In the prophylactic cohort we started administering DLI after a median time of 100 days (range 88–324) after HSCT and in the therapeutic cohort after a median time of 261 days (range 141–776 days). All patients were closely monitored for symptoms of GVHD according to the classification by the Mount Sinai Acute GVHD International Consortium [8]. We increased the DLI dose only in patients who tolerated the previous dose without any symptoms of GVHD and administered DLI monthly with a median number of eight infusions (range: 2–48) during a median time of 9 months.

### 2.3 Disease Monitoring

Disease specific genetic markers were detected by standard sequencing methods and used to monitor the presence of MRD. MRD was closely monitored by polymerase chain reaction and flow cytometry.

Donor chimerism was measured by short tandem repeat analyses from peripheral blood and bone marrow. In patients with malignant disease, the goal of treatment was complete donor chimerism. In nonmalignant diseases, achieving a stable mixed donor chimerism is generally sufficient, as it typically ensures patient well‐being and prevents the recurrence of disease‐related symptoms [9].

Bone marrow aspirates from patients with hematological diseases were analyzed morphologically to monitor blast counts. Patients with solid tumors received regular imaging according to standard guidelines. According to current treatment protocols, we defined hematological relapse morphologically if a bone marrow smear contained more than 5% of blasts [10].

### 2.4 Additional Therapy

Some of the patients received additional treatment with demethylating agents such as azacitidine. Others were treated with tyrosine kinase inhibitors such as ponatinib or sorafenib. By enhancing the former downregulated production of IL‐15 in AML blasts, sorafenib increases the therapeutic potential of DLI [11]. Some patients also received antibody treatment. Four patients were administered the antibody blinatumomab, which immunologically couples the CD3 receptor of T cells to CD19‐positive leukemia cells, inducing their apoptosis. Two patients were treated with inotuzumab ozogamicin, a monoclonal antibody directed against CD22.

Four patients received zoledronic acid simultaneously to each DLI dose. Based on the current state of research, this bisphosphonate has been shown to enhance the graft‐versus‐malignancy effect by promoting γδ T‐cell cytotoxicity [11, 12].

In the preemptive cohort, patients with ALL and a high MRD load received either zoledronic acid (*n* = 4) or blinatumomab (*n* = 3) or inotuzumab ozogamicin (*n* = 1) in addition to DLI. In another patient with ALL, we started with administering blinatumomab and switched to inotuzumab ozogamicin because of insufficient efficacy. We combined ponatinib with DLI in a patient with CML. In the prophylactic cohort, one patient received azacitidine and one zoledronic acid. In the therapeutic cohort, we administered DLI in combination with sorafenib (*n* = 2) or azacitidine with zoledronic acid (*n* = 1).

### 2.5 Statistical Analysis

Outcome variables of interest were response to DLI, course of donor chimerism, acute and chronic GVHD (aGVHD and cGVHD), GVHD‐free and relapse‐free survival (GRFS) and overall survival (OS). We used electronic medical records and archived files to collect clinical, laboratory, and demographic data. The patients' 3‐year GRFS and 5‐year OS were estimated by Kaplan–Meier survival analyses and log‐rank tests. The cumulative incidence of relapse was calculated using Gray's test. GRFS was calculated between the date of first DLI and date of relapse, graft failure, death or first signs of aGVHD, or cGVHD. Regardless of cause, OS was defined as interval from date of first DLI to date of death.

## 3 Results

### 3.1 Preemptive DLI

We treated 12 patients with preemptive DLI because of MRD positivity. Ten patients responded and achieved a complete molecular remission (83%) and maintained it over a median follow‐up period of 46.5 months. In the other two patients of this cohort, hematological relapse could not be prevented. Because of high MRD load in ALL patients, DLI administration was combined with either zoledronic acid or inotuzumab ozogamicin or blinatumomab in one patient each. In another patient with ALL, two antibodies were administered one after the other. Yet only a combination of DLI and inotuzumab ozogamicin resulted in complete donor chimerism and molecular remission. We successfully combined DLI with ponatinib in one patient with CML. Nevertheless, two patients progressed to a hematological relapse that led to death in one of them.

**FIGURE 1:**
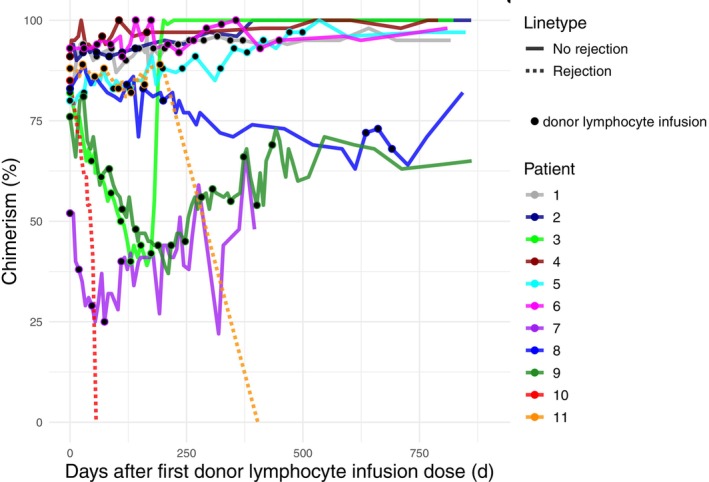
Course of donor chimerism in patients with genetic disease.

**FIGURE 2:**
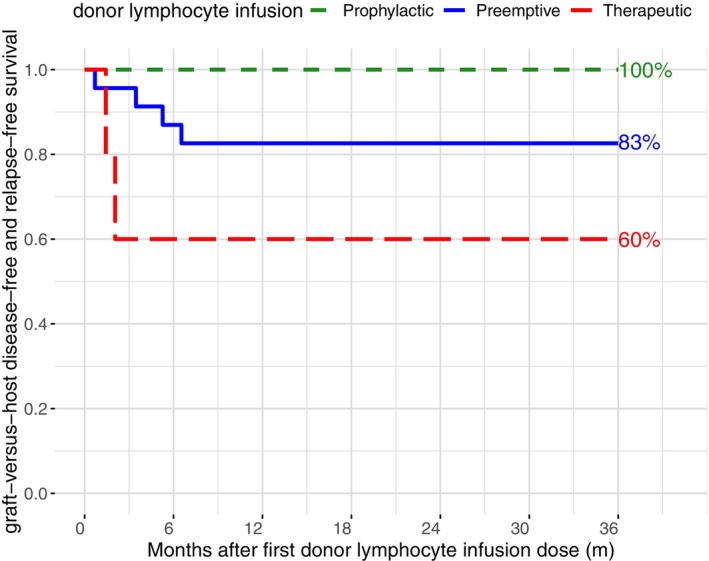
Graft‐versus‐host disease–free and relapse–free survival in patients of the preemptive, prophylactic, and therapeutic cohort.

**FIGURE 3:**
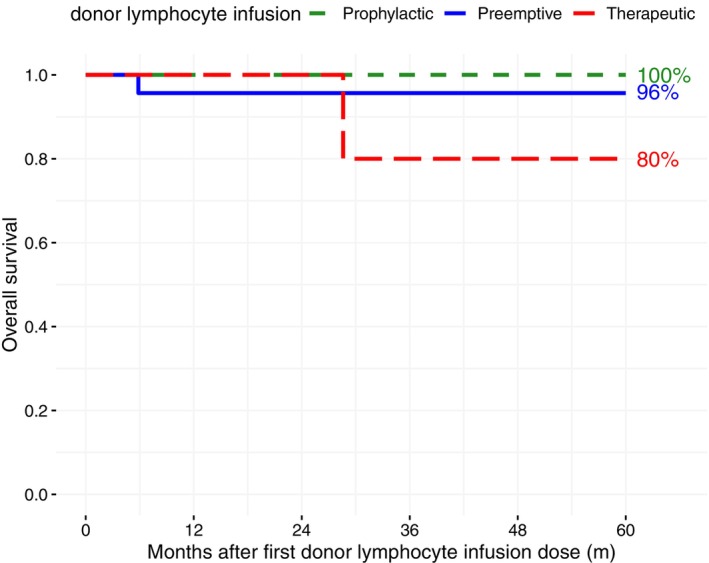
Overall survival in patients of the preemptive, prophylactic, and therapeutic cohort.

We treated 11 patients with genetic disease with preemptive DLI. All of them showed MC and were at risk for graft rejection. In total, nine patients (82%) were treated successfully. Six patients (55%) responded with increasing donor chimerism above 95%. Three patients developed stable MC at a median of 70%. The remaining two patients had a secondary graft failure and had to be retransplanted. Development of donor chimerism after HSCT is shown in Figure 1. The 3‐year GRFS for all patients treated with preemptive DLI was 83% (Figure 2) and the 5‐year OS was 96% (Figure 3).

### 3.2 Prophylactic DLI

We administered prophylactic DLI in six patients suffering from high‐risk malignancies. One patient with relapsed JMML received azacitidine in addition, and another patient with Ewing sarcoma received zoledronic acid additionally. As all patients of the prophylactic cohort are in CR, the safety of DLI administration is the highest priority. To prevent the occurrence of GVHD, we started at a lower median DLI dose than in the preemptive or therapeutic cohort and increased very carefully. Overall, all patients (100%) had a successful outcome without relapse or GVHD (Figure 2), and the 5‐year OS was 100% (Figure 3).

### 3.3 Therapeutic DLI

Four patients were treated with therapeutic DLI due to hematological relapse, with all patients showing a confirmed relapse of 5% blasts detected in peripheral blood. We administered DLI in addition to sorafenib in two patients with relapsed AML. Both patients showed a successful outcome and achieved complete molecular remission. The remaining two patients relapsed. The fifth patient of the therapeutic cohort suffered from recurrent autoimmune hemolytic crises and was successfully treated with DLI. In the therapeutic cohort, the GRFS was 60% (Figure 2) and the 5‐year OS 80% (Figure 3).

### 3.4 GVHD

We observed acute GVHD only in two patients (6%) of the preemptive cohort. One patient who was treated with DLI because of MC in genetic disease suffered from acute GVHD Grade I of the skin. We successfully treated him with methylprednisolone and extracorporeal photopheresis. Another patient with relapse in JMML developed GVHD grade II of the skin and intestine. He responded to our therapy with methylprednisolone and cyclosporine A. Both patients with GVHD could be cured in a short time period. However, both patients benefited from DLI therapy and responded with an increase of donor chimerism and CR, respectively. We observed no chronic GVHD in our patients.

## 4 Discussion

DLI remains the most widely used immunotherapeutic strategy to prevent or treat relapse following HSCT, particularly in hematological malignancies [13]. Numerous studies across both clinical and preclinical models have consistently demonstrated the efficacy of DLI in enhancing GVL and GVT effects. Although the sensitivity to DLI varies considerably between different malignant diseases, DLI has shown efficacy across a wide range of hematological malignancies [14]. Mixed donor chimerism in patients with malignant disease was found to be a significant risk factor for relapse. In contrast, however, complete donor chimerism in patients with nonmalignant disease is not necessary, as there are no advantages in transplant outcomes in comparison to patients with MC. However, a significant decline in donor chimerism in blood correlates with an increased risk of graft failure, and rejection may be prevented by timely DLI administration [15].

Overall, 82% of all patients included in this study responded to DLI administration. We report a GRFS of 83% in the preemptive cohort, 100% in the prophylactic cohort, and 60% in the therapeutic cohort. The OS was 96% in the preemptive cohort, 100% in the prophylactic cohort, and 80% in the therapeutic cohort. Compared to other studies about the efficacy of DLI after HSCT, we achieved similar to slightly better results in all categories [1, 2, 16]. With a median of 8 doses of DLI per patient, only a few centers perform infusions as frequently. The high number of DLI applications was due to the patients' particularly high risk of relapse. Since the DLI administrations were well tolerated, treatment was continued even beyond the recommended number to minimize the risk of relapse. Of course, it remains uncertain whether fewer doses might have been sufficient.

Furthermore, it should be noted that some of the patients received additional treatment as explained earlier. These co‐interventions may have influenced outcomes and should be considered when interpreting the results. Both blinatumomab and inotuzumab ozogamicin have been shown to induce deep remissions, suggesting that the therapeutic success observed may not be solely attributable to DLI. This is a clear limitation of our study, as using additional treatments makes it difficult to determine the specific contribution of DLI to the overall outcome. Still, additional treatment was given for ethical reasons and to minimize the risk of relapse, as it would have been inappropriate to withhold potentially beneficial therapy. Based on our clinical experience, the combination of DLI with other modalities appears to be the most effective approach, and we believe that this multimodal strategy has been key to achieving favorable outcomes.

We started at a median DLI dose of 1.0 × 10^5^ CD3 cells/kg in the preemptive cohort, 3.2 × 10^4^ CD3 cells/kg in the prophylactic cohort and 1.0 × 10^6^ CD3 cells/kg in the therapeutic cohort. For patients treated with DLI after haploidentical HSCT we started at a DLI dose of 1.0 × 10^4^ CD3 cells/kg. Our starting doses were lower than in comparable studies, as a higher dose is associated with an increased risk of GVHD [14, 16]. Moreover, there are currently no established guidelines for the administration of DLI in pediatric patients. According to the recently published practice recommendations for adults from the EBMT [14], a dose escalating regimen reduces the occurrence of GVHD in case of multiple DLI. Our practical experience aligned with these recommendations. In this study we increased DLI doses gradually in half‐log increments and continued with the administration only in patients who tolerated the previous dose without any symptoms of GVHD. To maximize the safety of our treatment, we did not exceed a DLI dose of 3.2 × 10^7^ CD3 cells/kg.

In our study, the median time between HSCT and DLI in the prophylactic cohort was 100 days (range 88–324 days), thus adhering to the commonly applied interval of around 90 days, which is known to reduce the risk of GVHD [17, 18]. This timing was chosen deliberately, as the occurrence of GVHD in the prophylactic setting should be strictly avoided.

In patients of the preemptive cohort with MRD, the interval between HSCT and DLI was 234 days (range 74–760 days). It showed greater variability and was in some cases shorter than 90 days. In these situations, the main goal was to prevent hematological relapse and the potential benefit outweighed the GVHD risk. In some patients, MRD positivity occurred later after HSCT, which led to a delayed decision to administer DLI. In patients with genetic disease of the preemptive cohort, the median interval between HSCT and DLI was generally the shortest with 88 days (range 23–311 days). DLI was administered after ensuring engraftment, typically 1–2 months posttransplant and following evaluation of donor chimerism [18]. In case of MC, DLI was administered at an early stage to prevent graft rejection.

As expected, the median time between HSCT and DLI was longest in the therapeutic cohort, since the indication for DLI in these patients was a manifest relapse, which occurs later after transplantation. The interval between HSCT and therapeutic DLI was 261 days (range 141–776 days).

Considering these aspects, we are able to report an extremely low rate of GVHD (6%) in all patients. Acute GVHD did not impact OS and there were no cases of chronic GVHD observed. Therefore, we consider DLI to be a safe, yet effective treatment option for patients in a preemptive, prophylactic, and therapeutic setting.

## Author Contributions

**Dinah Walther:** conceptualization, original draft preparation. **Jana Ernst:** conceptualization, patient recruiting. **Carola Wollenhaupt:** methodology, formal analysis. **Susan Wittig:** methodology, formal analysis. **Manuela Härtel:** methodology, formal analysis. **Grit Brodt:** patient recruiting. **Till Milde:** supervision. **Bernd Gruhn:** conceptualization, patient recruiting, original draft preparation, project administration. All authors have read and agreed to the published version of the manuscript.

## Funding

The authors have nothing to report.

## Ethics Statement

All procedures were in accordance with the ethical standards. The study has been approved by the Jena University Hospital Ethics Committee (2024‐3396).

## Conflicts of Interest

The authors declare no conflicts of interest.

## Data Availability

The data that support the findings of this study are available from the corresponding author upon reasonable request.
